# Shoulder posture and median nerve sliding

**DOI:** 10.1186/1471-2474-5-23

**Published:** 2004-07-28

**Authors:** Andrea Julius, Rebecca Lees, Andrew Dilley, Bruce Lynn

**Affiliations:** 1 Department of Physiology, University College London, Gower Street, London, UK

## Abstract

**Background:**

Patients with upper limb pain often have a slumped sitting position and poor shoulder posture. Pain could be due to poor posture causing mechanical changes (stretch; local pressure) that in turn affect the function of major limb nerves (e.g. median nerve). This study examines (1) whether the individual components of slumped sitting (forward head position, trunk flexion and shoulder protraction) cause median nerve stretch and (2) whether shoulder protraction restricts normal nerve movements.

**Methods:**

Longitudinal nerve movement was measured using frame-by-frame cross-correlation analysis from high frequency ultrasound images during individual components of slumped sitting. The effects of protraction on nerve movement through the shoulder region were investigated by examining nerve movement in the arm in response to contralateral neck side flexion.

**Results:**

Neither moving the head forward or trunk flexion caused significant movement of the median nerve. In contrast, 4.3 mm of movement, adding 0.7% strain, occurred in the forearm during shoulder protraction. A delay in movement at the start of protraction and straightening of the nerve trunk provided evidence of unloading with the shoulder flexed and elbow extended and the scapulothoracic joint in neutral. There was a 60% reduction in nerve movement in the arm during contralateral neck side flexion when the shoulder was protracted compared to scapulothoracic neutral.

**Conclusion:**

Slumped sitting is unlikely to increase nerve strain sufficient to cause changes to nerve function. However, shoulder protraction may place the median nerve at risk of injury, since nerve movement is reduced through the shoulder region when the shoulder is protracted and other joints are moved. Both altered nerve dynamics in response to moving other joints and local changes to blood supply may adversely affect nerve function and increase the risk of developing upper quadrant pain.

## Background

Non-specific arm pain (NSAP), often called repetitive strain injury, describes the common problem of upper limb pain and functional impairment without objective physical findings. The contributing factors to the development of NSAP are not fully understood but ergonomic guidelines commonly suggest that good upper body posture protects against NSAP (e.g. [1]). In a study of 485 NSAP patients, shoulder protraction and forward head position were reported in a majority of patients (78% and 71% respectively) [2]. Poor upper body posture (e.g. rounded shoulders, head forward) has also been reported to increase the incidence of neck and shoulder pain [3]. The possible mechanisms leading to pain in patients with postural malalignment have not been examined in any detail. The painful symptoms often associated with NSAP suggest a minor neuropathy involving at least in part the median nerve [4].

Together, shoulder protraction, forward head position and flexion of the trunk form the main components of slumped sitting. The present study examines the effects of each of these components on longitudinal sliding of the median nerve using high frequency ultrasound imaging in asymptomatic normal subjects. In addition, the effects of sustained protraction on nerve sliding through the shoulder region are examined.

## Methods

### Ultrasound imaging

Longitudinal nerve movement was measured using high frequency ultrasound imaging, as previously described by Dilley et al. [5,6]. A Diasus ultrasound system (Dynamic Imaging, Livingston, Scotland, UK) was used to collect sequences of ultrasound images at 10 frames/second for 50 to 70 seconds, running at 10–22 MHz and using a 26 mm linear transducer. A cross-correlation algorithm was used to determine relative movement between adjacent frames in sequences of images [5]. The maximum correlation coefficient (r) was calculated for each pixel shift determining the relative movement between frames. To account for probe movement the same method was employed on deep stationary structures (eg. bone or interosseous membrane) and the result subtracted from the nerve excursion values.

### Subject details

Fourteen healthy subjects, 5 male and 9 female, aged 25–38 years (mean = 32 years) were screened to exclude upper limb or cervical spine pathologies, rheumatological or neurological conditions. In each subject the nerve bed length was estimated, from the C6 spinous process to the tip of the index finger (mean = 97.0 cm (SD, 5.9)), and used to normalise the ultrasound transducer position between individuals. All measurements were taken from the right upper limb only.

### Set-up and procedure

The median nerve was imaged in longitudinal section in the forearm during forward head position, trunk flexion and protraction and in the forearm and upper arm during contralateral neck side flexion (CNSF). Each movement was repeated three times, including some reverse trials.

#### Forward head position

Each subject (n = 8) was imaged in the proximal forearm whilst positioned upright on a chair fitted with a back and head support, hips and knees at 90° flexion, and the trunk fixed with Velcro strapping. The right upper limb was strapped to a Perspex plate in 90° flexion and 20° abduction at the glenohumeral joint, with the elbow fully extended, 45° forearm supination, and the wrist, hand and fingers in neutral. An active forward head position movement was performed, which included lower cervical spine flexion and upper cervical spine extension.

#### Trunk flexion

Each subject (n = 8) was imaged in the proximal forearm whilst positioned upright on a chair, with hips and knees at 90 degrees flexion. The right upper limb was positioned as for the forward head position trials. The subject was taught to actively flex their trunk whilst posteriorly tilting their pelvis.

#### Protraction

Each subject (n = 13) was imaged at two locations in the forearm and positioned as for the forward head position. The distal upper arm was imaged in three of the 13 subjects. For each trial the shoulder girdle was passively protracted from neutral (i.e. the scapulothoracic joint in neutral) by sliding the Perspex plate supporting the arm on an adjustable table. In three of these subjects, additional data was also obtained during ultrasound imaging. A potentiometer attached using strong thread to the acromion process allowed measurement of the amount of protraction. Protraction data was captured on to a PC and synchronised offline to the recorded ultrasound sequence.

In four subjects, good quality images of the median nerve within the upper arm could be obtained. In the majority of subjects, it was difficult to acquire good quality images because of dense tissue overlying the nerve that reduced the image quality. From these images, nerve trunk bowing was measured in the distal upper arm with the shoulder girdle in the neutral and protracted positions. The maximum deviation of the nerve from a straight line across single ultrasound frames was measured offline in both positions and the difference used as a measure of additional bowing. Repeat trials were averaged.

#### Contralateral neck side flexion

Each subject (n = 11) was imaged in the distal forearm and distal upper arm, whilst lying supine with the right upper limb abducted to 90° at the glenohumeral joint. Ninety degree abduction at the glenohumeral joint rather than 90° glenohumeral flexion was used, so that the present data could be related to previous work [6] which has shown that median nerve movements can be reliably measured with the glenohumeral joint abducted to 90°. The examined limb was fixed to a Perspex plate using Velcro strapping with the elbow extended, forearm supinated and the wrist, hand and fingers in neutral.

The head was supported on a movable plate, with the centre of rotation positioned at the C7 spinous process. In each subject, the neck was passively moved to 35° CNSF with (a) the scapulothoracic joint in neutral (i.e. relaxed lying in supine) and (b) in full protraction. This movement was repeated several times. In four subjects, a potentiometer attached to the plate allowed continuous measurement of the angle of CNSF. Joint angle data was captured on to a PC and synchronised offline to the recorded ultrasound sequence.

### Subject movement measurements

**Figure 1:**
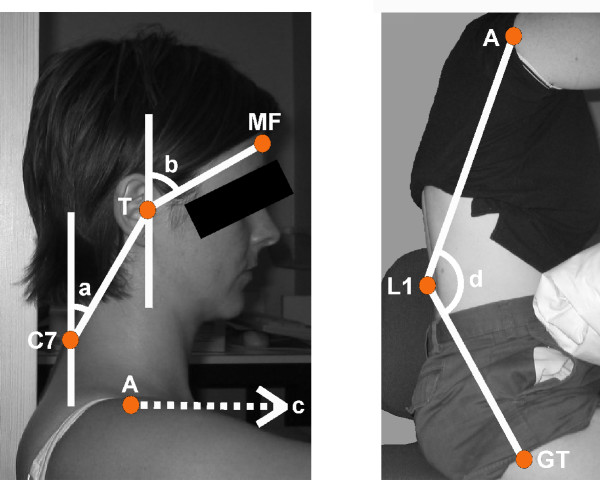
Summary of subject movement measurements. (a) Lower cervical spine flexion: the change in angle from a line extending from C7 to the tragus of the ear (T) and a vertical line through C7. (b) Upper cervical spine extension: the change in angle from a line extending from the tragus to the mid forehead (MF) and a vertical line through the tragus. (c) Protraction: the change in distance of the acromion (A) along the horizontal axis. (d) Trunk flexion: the change in angle from two lines extending from L1 to the acromion (A) and L1 to the greater trochanter (GT).

For each procedure the range of movement was determined from pictures obtained using a digital camera. Changes in joint angle and distance for each movement were determined from skin surface markers and measured using either CorelDraw (Kodak Digital Science, USA) or "tpsDig" (F. James Rohlf, Department of Ecology and Evolution, State University of New York). Measurements for the individual components of slumped sitting are summarised in figure 1. The posterior-anterior shift of the acromion was used as a measure of protraction during CNSF.

### Strain calculations

Strain is defined by the difference in the amount of elongation that occurs at two points along a nerve divided by the distance between these two points. In practice strain was determined by using regression lines fitted to plots of nerve movement against the distance along the arm. Note that the strain estimates represent the additional strain produced by the movement rather than the total nerve strain.

### Statistical analysis

Comparisons of nerve movement and strain in scapulothoracic neutral and protraction during CNSF were performed using paired t-tests.

## Results

### Median nerve movements in the arm in response to components of slumped posture

#### Forward head position

Moving the head forward while maintaining the shoulder and trunk position was tested in 8 subjects. This movement produced no detectable median nerve excursion in the forearm, the average trend being a movement of 0.1 mm (SEM, 0.02) occurring in a proximal direction. The repeat measure variability within subjects was very low, with a standard deviation ranging from 0–0.2 mm (mean = 0.1 mm). The mean change in the angle of lower cervical spine flexion and upper cervical spine extension was 23.6° (SD, 2.8) and 2.9° (SD, 1.9) respectively.

#### Trunk flexion

Trunk flexion also produced minimal median nerve excursion with a mean over 8 subjects of 0.1 mm (SEM, 0.1) proximal movement. The mean change in the angle of trunk flexion was 19.7° (SD, 4.7).

#### Shoulder protraction

**Figure 2:**
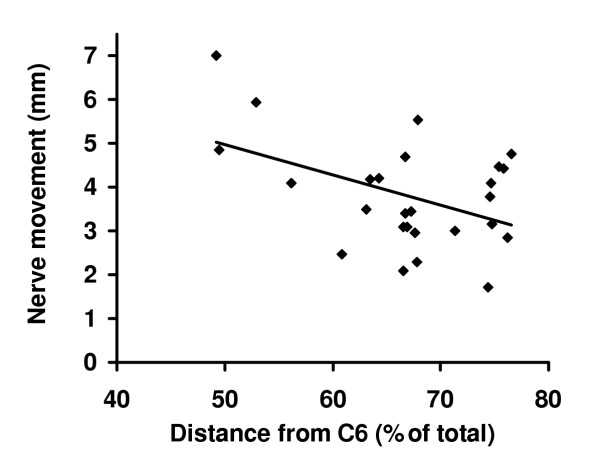
Individual nerve excursion values at sites in the upper arm and forearm for thirteen subjects produced by protraction. Each point is the average of three individual trials. Distance along the arm has been expressed as percentage of total distance from C6 spinous process to the tip of the index finger. A regression line has been fitted to the data.

In 13 subjects the median nerve moved in a proximal direction during shoulder protraction with more movement at proximal locations (mean in forearm = 3.5 mm (SEM, 0.3), mean in upper arm = 5.9 mm (SEM, 0.6)) (figure 2 [see additional file 1 for ultrasound sequence of median nerve sliding in the forearm]). The mean extent of scapular anterior translation was 38.3 mm (SD, 13). The additional strain on the median nerve was 0.7% (SEM, 0.3), given by the slope of the regression of nerve movement against distance along the arm (Figure 2).

**Figure 3:**
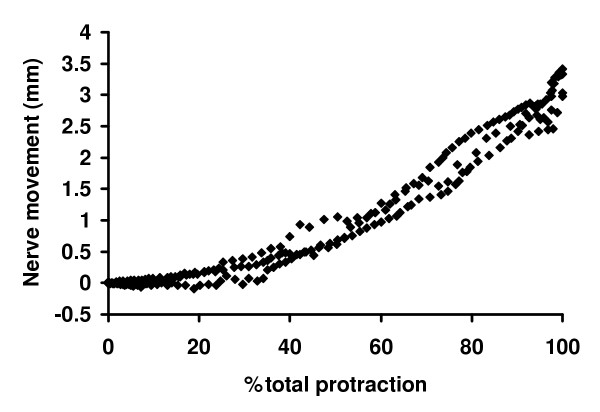
Nerve movements in forearm plotted against protraction (expressed as a percent of the total movement) (n = 3). Total protraction movements ranged from 40 to 95 mm. Each curve is a single trial from one of 3 subjects. Note the initial delay in nerve movement.

Median nerve excursion was measured in 3 subjects with simultaneous measurement of protraction. The results revealed an initial delay of 6.5–33.0 mm (mean = 17.0 mm; equivalent to 15.8–34.0% (mean = 23.7%) of the total protraction) before significant nerve movement occurred (see figure 3). After the initial delay, nerve movement was proportional to the extent of protraction.

**Figure 4:**
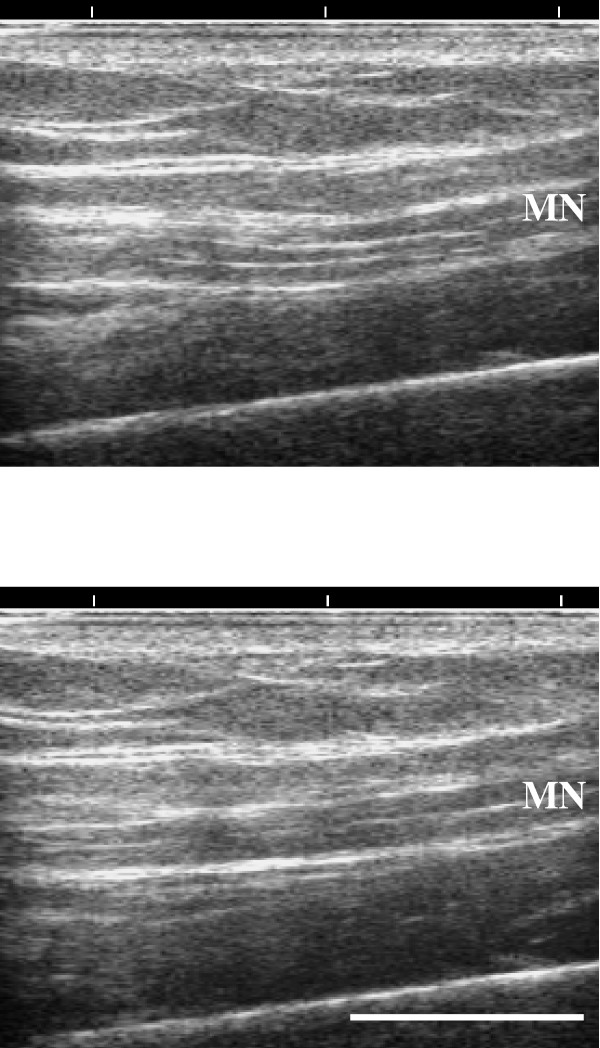
Bowing of the median nerve. Ultrasound images of the median nerve in the distal upper arm (upper) with the shoulder girdle in neutral and (lower) protracted. Note substantial bowing with the shoulder girdle in the neutral compared to protracted position. Bar = 10 mm.

In three of four subjects, nerve bowing was observed in the upper arm with the shoulder girdle in the neutral test position. The maximum nerve course deviation from a straight line with the shoulder girdle in neutral compared to protraction was approximately 0.5 mm in all three subjects over the length of the ultrasound transducer, (26 mm). The nerve straightened during protraction (figure 4).

### Median nerve movement in the arm in response to contralateral neck side flexion (CNSF) with or without shoulder protraction

**Figure 5:**
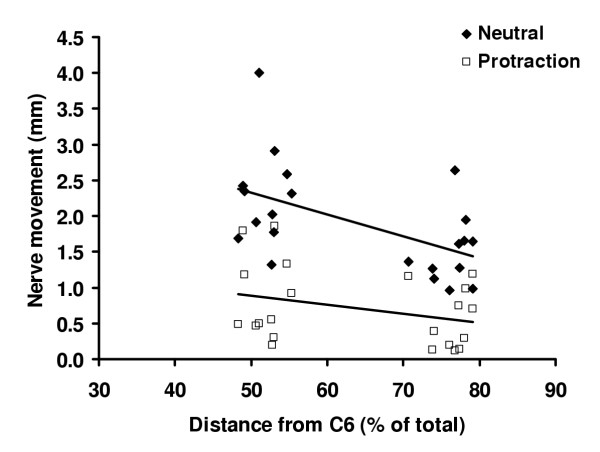
Individual nerve excursion values (mm) in response to CNSF for eleven subjects in scapulothoracic neutral and in protraction. Each point is the average of three individual trials. Distance along the arm has been expressed as percentage of total distance from C6 spinous process to the tip of the index finger. A regression line has been fitted to the data.

In 11 subjects the median nerve moved in a proximal direction during 35° CNSF when the scapulothoracic joint was in neutral, as reported previously [6]. The movement increased at the more proximal location (scapulathoracic neutral, mean in upper arm = 2.3 mm (SEM, 0.2) and forearm = 1.5 mm (SEM, 0.2)). With the shoulder protracted, there was a 60% reduction in nerve movement in both upper arm and forearm locations (p < 0.05 for both locations) (mean in upper arm = 0.9 mm (SEM, 0.2) and forearm = 0.6 mm (SEM, 0.1)) (figure 5). The mean extent of protraction was 48.0 mm (SEM, 4.3). The additional strain on the median nerve was 0.3% (SEM, 0.1) in scapulothoracic neutral. There was a significant reduction in strain in protraction (0.1% (SEM, 0.1); p < 0.05, paired t-test).

**Figure 6:**
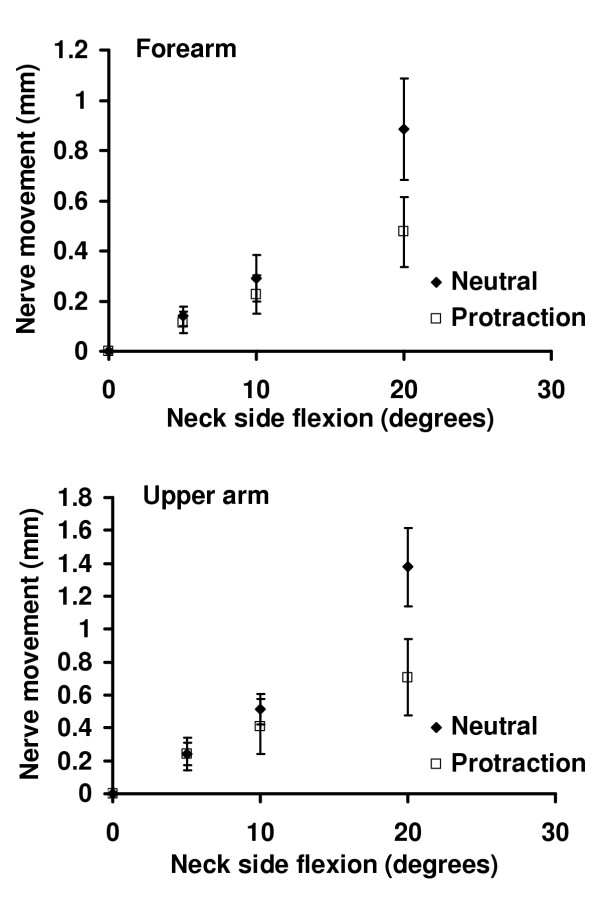
Nerve movements in the forearm (upper) and upper arm (lower) plotted against neck angle with the shoulder in scapulothoracic neutral and protraction. Each data point is the average of 4 subjects. Note the absence of a delay in nerve movement in protraction compared to scapulothoracic neutral. Error bars = SEM.

Median nerve excursion was measured in 4 subjects with simultaneous measurement of CNSF. The results revealed no obvious delay in the onset of nerve movement in protraction compared to scapulothoracic neutral. Despite less movement in protraction, the pattern of nerve movement mimicked that observed in scapulothoracic neutral (figure 6).

Nine of 11 subjects reported paraesthesia in the distribution of the median nerve dermatone once the shoulder girdle was sustained in protraction. The onset of symptoms ranged from 1 to 4 minutes. Symptoms disappeared when the shoulder was repositioned in scapulothoracic neutral.

## Discussion

### Direct effects of the components of slumped sitting on median nerve movement

Nerves are designed to slide and stretch to accommodate joint movement. Using the method of Dilley et al. [5,6], median nerve sliding was examined during the individual components of slumped sitting. Both forward head position and trunk flexion produced only minimal nerve movement in the forearm. The only examined component to produce substantial nerve movement was shoulder protraction. The median nerve strain in the forearm with protraction was 0.7%, which was well below the limits that cause changes to nerve function (reviewed in Grewel et al. [7]).

As the shoulder girdle is protracted there is a delay in nerve movement, which is followed by a steady increase in nerve excursion. During this initial toe region the median nerve appears bowed in the upper arm. The nerve trunk appears to straighten as the range of protraction progresses. It therefore seems that with the upper limb in scapulothoracic neutral and the glenohumeral joint in 90° flexion, the median nerve is unloaded. If this is the case, the strain value of 0.7% will represent the total strain.

### Effects of protraction on the transmission of median nerve movement through the shoulder region

The results for CNSF provide evidence for a possible restriction within the shoulder region during shoulder protraction. With the shoulder protracted there was a 60% reduction in the transmission of nerve movement through the upper limb. Consistent with a reduction in movement, there was also significantly less strain in the forearm. The possibility that the nerve becomes unloaded when the shoulder is protracted is unlikely since it had been found that protraction itself causes some median nerve stretch. In addition, there was no obvious delay in nerve movement in response to CNSF (Figure 6). The evidence for a restriction is consistent with previous suggestions that shoulder protraction may cause a neurovascular impingement within the shoulder region resulting in pain [2,8]. This suggestion was further supported by the experience of paraesthesia within the median nerve distribution during sustained protraction in 82% of subjects. These symptoms indicate the presence of a vascular restriction, which in turn affects neural function.

Scapular protraction is a complicated movement, often resulting in the combined movement of numerous other structures within the shoulder girdle, including anterior displacement of the head of the humerus. It is therefore difficult to establish the precise cause of a neurovascular entrapment. Shortening of pectoralis minor and the downward displacement of the coracoid process might affect sliding of the cords of the brachial plexus. Alternatively, elevation of the first rib during full protraction (due to its soft tissue attachments with surrounding structures) might reduce the space between the clavicle and the first rib, restricting nerve sliding.

### Clinical significance

The components of slumped sitting (i.e. forward head position, trunk flexion and protraction) are associated with poor posture [2,9-11], and are often adopted by office workers. Shoulder protraction is the only component of this posture to tension the median nerve, although the level of nerve strain in the forearm with the shoulder at 90° flexion and elbow extension, is not sufficiently high to result in direct neural injury.

Problems are more likely to result from local effects of shoulder protraction on the chords of the brachial plexus. The present study shows that protraction restricts nerve sliding through the shoulder region. Most subjects also experienced paraesthesia when maintaining shoulder protraction plus elbow extension and shoulder abduction. Therefore, sustained shoulder protraction may place the median nerve at enhanced risk of injury and possibly cause a vascular compromise. This may in turn explain for the trend that a high number of NSAP patients have poor shoulder posture. (e.g. [2]).

## Conclusions

The direct effects of slumped sitting on median nerve strain are not sufficient to alter nerve function. However, shoulder protraction does appear to restrict nerve sliding, and prolonged protraction leads to pareasthesias.

## Competing interests

None declared.

## Authors' contributions

AJ and RL participated in the study design, data collection, analysis and manuscript preparation. AD participated in the study design, analysis and manuscript preparation. BL participated in the study design and manuscript preparation. All authors read and approved the final manuscript.

## Pre-publication history

The pre-publication history for this paper can be accessed here:



## Supplementary Material

Additional File 1 **Median nerve sliding in the forearm during protraction.** Ultrasound sequence of median nerve sliding in the distal forearm during protraction. The subject was imaged with the limb in 90° flexion and 20° abduction at the glenohumeral joint and elbow neutral. The median nerve can be seen to slide in a proximal direction as the shoulder is protracted. In a repeat of the sequence the nerve movement is tracked using cross-correlation analysis (yellow plus sign). The total nerve movement was 4.70 mm. Click here for file
